# Dragon Fruit Peel Waste (*Hylocereus undatus*) as a Potential Ingredient for Reducing Lipid Peroxidation, Dietary Advanced Glycation End Products, and Starch Digestibility in Cookies

**DOI:** 10.3390/antiox12051002

**Published:** 2023-04-26

**Authors:** Siriwan Chumroenvidhayakul, Thavaree Thilavech, Mahinda Abeywardena, Sirichai Adisakwattana

**Affiliations:** 1 Phytochemical and Functional Food Research Unit for Clinical Nutrition, Department of Nutrition and Dietetics, Faculty of Allied Health Sciences, Chulalongkorn University, Bangkok 10330, Thailand; siriwan.chu112@gmail.com; 2 Department of Food Chemistry, Faculty of Pharmacy, Mahidol University, Bangkok 10400, Thailand; 3 CSIRO Health & Biosecurity, Kintore Avenue, Adelaide, SA 5000, Australia; mahinda.abeywardena@csiro.au

**Keywords:** dragon fruit peel, cookie, starch digestion, advanced glycation end products

## Abstract

Excessive consumption of cookies has been linked to harmful health outcomes owing to the presence of refined carbohydrates and heat-induced toxicants including end products of lipid peroxidation and dietary advanced glycation end products (dAGEs). To address this issue, this study explores the addition of dragon fruit peel powder (DFP), which is rich in phytochemicals and dietary fibers, to cookies as a potential solution to mitigate their adverse effects. The results indicate that adding DFP at 1%, 2%, and 5% *w*/*w* of raw cookie dough significantly improves the total phenolic and betacyanin contents and antioxidant activity, as evidenced by increased ferric-reducing antioxidant power. DFP incorporation also led to reductions in malondialdehyde and dAGEs (*p* < 0.05). Furthermore, the starch digestibility, hydrolysis index, and predicted glycemic index were all reduced in the presence of DFP, with the latter estimate being due to the higher content of undigested starch. Incorporating DFP in cookies resulted in significant changes in their physical properties, including texture and color. However, sensory evaluation indicates that the overall acceptability of the cookies was not negatively impacted by the addition of up to 2% DFP, suggesting that it is a viable option for enhancing the nutritional value of cookies without compromising their palatability. These findings suggest that DFP is a sustainable and healthier ingredient that can improve the antioxidant capacity of cookies while also mitigating the harmful effects of heat-induced toxins.

## 1. Introduction

Bakery products, including cookies, are a popular food choice worldwide because of their accessibility and delicious taste. Nevertheless, their nutritional value has been extensively researched, revealing certain concerns [1,2]. Moreover, their high content of refined carbohydrates, simple sugars, and fat, combined with low dietary fiber content, poses a significant risk to human health [3]. In addition, cookies can undergo non-enzymatic reactions and oxidation during baking, which can lead to the formation of glycated molecules [4]. These molecules can then undergo further chemical transformations, resulting in the production of toxic lipid peroxidation products (such as malondialdehyde (MDA)), and dietary advanced glycation end products (known as dAGEs) [1,4,5]. Remarkably, consuming cookies with a high glycemic index and heat-generated toxicants has been linked to the development of hyperglycemia and increased levels of circulating oxidized low-density lipoprotein, MDA, and AGEs in human serum and tissues [5,6]. These harmful compounds can accelerate the progression of several chronic diseases such as inflammation, cancer, atherosclerosis, diabetes, and glomerulosclerosis [5,6,7]. To address this issue, minimizing the glycemic index and decreasing the formation of MDA and dAGEs in cookies can help alleviate their potential negative impact on human health.

In recent years, researchers have emphasized the significance of managing the glycemic index and mitigating the formation of heat-induced toxicants, such as lipid peroxidation products and dAGEs, in food formulations. One promising strategy to attain these objectives is by reformulating food products using dietary fibers and phytochemicals derived from plants, which possess potent antioxidant properties [3,8,9]. For example, cookies fortified with apple flowers have been shown to effectively prevent the formation of reactive dicarbonyl compounds, such as methylglyoxal (MGO), and fluorescent AGEs, owing to the presence of flavonoids that exhibit remarkable scavenging and metal-chelating capabilities. These findings provide a plausible explanation for the observed MGO-scavenging and antiglycative properties of the cookies in both fructose and glucose BSA models [10,11]. In addition, the inclusion of dietary fibers and green tea polyphenols has been found to significantly reduce the levels of available glucose and acrylamide content in baked starchy food models [12]. The mechanism behind the decrease in starch digestibility can be attributed to the modification of starch granule microstructure, induced by green tea polyphenols, whereas dietary fibers create a physical barrier that impedes the access of amylolytic enzymes [13,14]. These findings highlight the potential of using natural ingredients as an alternative approach to enhance the nutritional value of cookies while mitigating their potentially harmful effects on human health.

Dragon fruit, scientifically named *Hylocereus undatus*, is a tropical fruit crop widely grown and consumed in Thailand. Although the flesh of the dragon fruit provides a significant source of vitamins, minerals, and dietary fibers, the peel, which accounts for 22% of the fruit’s weight, is typically discarded after consumption, despite its potential nutritional and functional benefits [15]. However, recent scientific studies have discovered that dragon fruit peels contain significant amounts of bioactive compounds such as betacyanins, phenolic compounds, and dietary fibers. These compounds exhibit a range of biological properties, including antioxidant, antidiabetic, antihyperlipidemic, and anticancer activities [16,17]. As a result of these benefits, dragon fruit peels are now considered a by-product with potential for use as a dietary fiber enhancer, natural colorant, and antioxidant in various food products. Interestingly, our prior investigation has established that the incorporation of dragon fruit peel powder (DFP) into various flour types led to a decline in starch digestibility, alteration of gelatinization enthalpy, and pasting properties, which were attributed to the presence of dietary fibers, phenolic compounds, and betacyanins [18]. In line with these findings, the aim of the present study was to investigate the effect of incorporating DFP into wheat cookies on starch digestibility, predicted glycemic index (pGI), and heat-induced toxicants. Additionally, the study evaluated the antioxidant activity, physical characteristics, and sensory attributes of DFP cookies, expanding the potential applications of dragon fruit peel as a functional food ingredient. The outcomes of this study have important implications for utilizing fruit waste, particularly dragon fruit peel, as a more sustainable and health-promoting material in the development of novel and nutritionally improved food products.

## 2. Materials and Methods

### 2.1. Materials

Dragon fruit peels were sourced from Ramathibodi Hospital in Bangkok, Thailand. The cookie ingredients used were commercially available from a supermarket. The following reagents and chemicals were purchased from Sigma-Aldrich Chemical Co. Ltd. (St. Louis, MO, USA): Folin–Ciocalteu reagent, betanin (betanidin 5-β-D-glucopyranoside), TPTZ (2,4,6-tripyridyl-s-triazine), 2,6-di-tert-butyl-4-methylphenol (BHT), 2-thiobarbituric acid (TBA), malondialdehyde tetrabutylammonium salt (MDA), pepsin from porcine gastric mucosa powder (250 U/mg), α-amylase type VI-B from porcine pancreas (15.8 U/mg), pancreatin from porcine pancreas, methylglyoxal (40% aqueous solution), 5-methylquinoxaline (5-MQ), and *o*-phenylenediamine (OPD). Amyloglucosidase from *Aspergillus niger* was purchased from Roche Diagnostics in Indianapolis, IN, USA.

### 2.2. Preparation of Dragon Fruit Peel Powder (DFP)

The DFP was prepared in accordance with a previously published method [18]. Fresh dragon fruit peels were first carefully washed, and the epicarp was removed. The peels were subsequently dried in a hot air oven for 12 h at a temperature of 60 °C. Once dried, the peels were ground and sieved through a No. 40 sieve to obtain the DFP. The powder was then stored in polyethylene foil bags at a temperature of −20 °C until further use. The characteristics of the DFP used in this study were previously described in our published research [18]. The proximate composition of DFP consisted of 70.85% carbohydrates, including 65.17% dietary fibers and 5.68% available carbohydrates. The remaining components were 15.91% ash, 6.37% protein, 5.81% moisture, and 1.06% fat. 

### 2.3. Preparation of Cookies

To prepare the cookie dough, all ingredients (presented in Table 1) were mixed according to the recipe by Mudgil et al. (2017) [19], with some modifications. The DFP was added to the dough at varying concentrations of 0% (control), 1%, 2%, and 5% (*w*/*w*) of the weight of raw cookie dough. The dough was subsequently baked at a temperature of 170 °C for a duration of 13 min. After baking, the cookies were allowed to cool to room temperature and stored under dry conditions at 25 °C in an aluminum foil bag for further analysis.

### 2.4. Determination of Phytochemical Compounds and Antioxidant Activity

In order to evaluate the active compounds and antioxidant activity, one gram of ground cookie from each sample was extracted with 20 mL of distilled water and continuously stirred in a shaker at 100 rpm for 3 h. The resulting mixture was then centrifuged at 2000× *g* for 15 min at 4 °C to collect the supernatant, which was filtered through Whatman No. 1 filter paper and stored in a dark room. The extracted cookie solution was used to determine the total phenolic content (TPC), betacyanin content (TBC), antioxidant activity (ferric-reducing antioxidant power (FRAP)), and lipid oxidation.

The TPC of cookies was determined according to Adisakwattana et al. [20]. In brief, the extracted cookie solution (50 μL) was mixed with 50 μL of a 10-fold dilution of Folin–Ciocalteu reagent and incubated at room temperature for 5 min. Then, 50 μL of 10% (*w*/*v*) Na_2_CO_3_ was added and further incubated for 60 min. The absorbance was read at 750 nm, and the results were expressed as mg gallic acid equivalent per 100 g cookie.

The TBC in cookies was determined according to the method previously described by Chumroenvidhayakul et al. [18]. The absorbance of the extracted cookie solution was measured at 538 nm, and the amount of TBC was calculated using a standard curve of betanin. The TBC was expressed as mg betanin equivalent per 100 g cookie.

To assess antioxidant activity, the FRAP assay was performed according to a previous publication [21]. Fresh FRAP reagent was prepared in the ratio of 10:1:1 of 0.3 M acetate buffer (pH 3.6), 10 mM TPTZ solution in 40 mM HCl, and 20 mM FeCl_3_, respectively. Then, the cookie solution (10 μL) was mixed with 90 μL of FRAP reagent and incubated for 30 min at room temperature. The absorbance was read at 595 nm, and the FRAP value was expressed in μmol FeSO_4_ equivalent per 100 g of cookie.

### 2.5. Determination of Lipid Peroxidation

The level of lipid peroxidation in cookies was measured according to a previous report [22], with modification based on the formation of thiobarbituric acid reactive substances (TBARSs). Briefly, the extracted cookie solution (200 μL) was mixed with 200 μL of 10% (*w*/*v*) trichloroacetic acid and 30 μL of 50 mM BHT. The mixture was centrifuged at 10,845× *g* at 4 °C for 15 min. The supernatant (200 μL) was mixed with 0.67% (*w*/*v*) TBA. The mixture was heated at 95 °C for 10 min and allowed to cool down at room temperature. The absorbance was read at a wavelength of 532 nm, and the results were calculated against the standard curve of MDA. The results were expressed as nmol MDA equivalent per 1 g cookie.

### 2.6. Determination of Released Glucose under Simulated Gastrointestinal Digestion

The glucose release from the cookie during simulated digestion was determined following the protocol described by Chumroenvidhayakul et al. [18]. Briefly, 500 mg of ground cookie or glucose (used as a reference compound) was mixed with 1 mL of artificial saliva solution (250 U/mL porcine amylase in 0.2 M carbonate buffer, pH 7) for 15–20 s, followed by the addition of 5 mL of pepsin suspension (1 mg/mL) in 0.02 M HCl (pH 2). The mixture was incubated in a water bath shaker (100 rpm) at 37 °C. After 1 h, the reaction was neutralized by adding 5 mL of 0.02 M NaOH and 25 mL of 0.2 M sodium acetate buffer (pH 6), and intestinal digestion was initiated by adding 5 mL of the enzyme mixture containing pancreatin (2 mg/mL) and amyloglucosidase (28 U/mL) in 0.2 M acetate buffer, pH 6. The mixture was further incubated at 37 °C in a water bath shaker (100 rpm), and samples were collected at 0, 20, 30, 60, 90, 120, and 180 min. The digesta fluid was immediately heated at 100 °C and centrifuged (10,845× *g*, 4 °C for 15 min), and the glucose concentration in the resulting supernatant was determined using a glucose oxidase kit (HUMAN GmbH, Wiesbaden, Germany). The percentage hydrolysis index (HI) and the predicted glycemic index (pGI) were calculated using the following equations:

HI = Area under the curve of released glucose from cookies/Area under the curve of standard glucose (reference) × 100
(1)

pGI = 39.71 + 0.549 HI
(2)

### 2.7. Determination of Starch Fraction

The total starch content (TS) in cookies was determined following a previous method [23], with slight modifications. In brief, 50 mg of cookie sample was mixed with 6 mL of 2 M KOH and incubated at room temperature for 1 h. The pH was then adjusted to 4.75 using concentrated HCl, and 3 mL of 0.4 M sodium acetate buffer was added. The mixture was further incubated at 60 °C for 45 min in a water bath shaker (100 rpm). After the mixture was heated to 100 °C, it was centrifuged at 10,845× *g* for 5 min. The glucose concentration was determined using a glucose oxidase kit. The total starch content was calculated by multiplying the glucose concentration by 0.9 and expressed as g/100 g sample. The rapidly digestible starch (RDS) was calculated as the difference between the glucose released at 20 min (G20) and free glucose (G0) after in vitro digestion, while the slowly digestible starch (SDS) was calculated as the difference between the amount of glucose measured at 120 min (G120) and 20 min. The amount of resistant starch (RS) was defined as the amount of glucose that remained undigested after 120 min. The equations used for calculating starch content are presented below:

RDS (%) = (G20 − G0)/TS × 100
(3)

SDS (%) = (G120 − G20)/TS × 100
(4)

RS (%) = (TS − (RDS + SDS))/TS × 100
(5)

where G0 = glucose content after 0 min of digestion; G20 = glucose content after 20 min of digestion; G120 = glucose content after 120 min of digestion; TS = total starch content.

### 2.8. Determination of Dietary Advanced Glycation End Products (dAGEs)

The fluorescent dAGEs in cookies were determined in accordance with a previous method [10]. Ground cookies weighing 250 mg were extracted using 4.75 mL of a buffer solution consisting of 0.05% Tween-20, 1% SDS, 5% β-mercaptoethanol, and 50 mM Tris-HCl at pH 7.4. The mixture was incubated at room temperature with shaking at 100 rpm for 12 h. Following extraction, the sample was centrifuged at 1904× *g* for 5 min, and the supernatant was analyzed using a spectrofluorometer (Perkin Elmer, Waltham, MA, USA) at excitation and emission wavelengths of 355 nm and 460 nm, respectively.

### 2.9. Determination of Methylglyoxal (MGO) Content

The MGO content in cookies was conducted following the method by Thilavech et al. (2016) [24], with some modifications. Briefly, five grams of ground cookies was extracted by sonication in 50 mL of 50% (*v*/*v*) methanol for 1 h. After centrifugation at 3808× *g* for 15 min, the supernatant was filtered through Whatman No. 1 filter paper. The filtrate was then subjected to concentration by rotary evaporation (Buchi, Switzerland) at 55 °C, and the extracted sample was redissolved in 1.5 mL of 50% (*v*/*v*) methanol in distilled water. Next, 900 μL of the extracted sample was mixed with 300 μL OPD (20 mM) and incubated at 37 °C for 24 h. The solution was then centrifuged at 10,845× *g* at 4 °C for 15 min. The determination was carried out by high-performance liquid chromatography (HPLC) using an LC-10 AD pump, an SPD-10A UV-VIS detector, and an Inersil-ODS3V C18 column (150 × 4.6 mm i.d.; 5 μm particle size) as the stationary phase. An isocratic program was conducted with 50% (*v*/*v*) methanol in distilled water as the mobile phase and a constant flow rate of 1 mL/min. The injection volume was 10 μL, and the absorbance was recorded at 315 nm. The total running time was 14 min, and the internal standard used was 5-methylquinoxaline in methanol (0.06%, *v*/*v*). The amount of MGO was calculated by comparing it to the standard curve of MGO.

### 2.10. Physical Properties of Cookies

Prior to the color measurement, the colorimeter (ColorFlex 45/0-HunterLab, Hunter Associates Laboratory, Inc., Reston, VA, USA) was calibrated using black and white standards. The color values were expressed using the CIE color scales, specifically L* for lightness (where 0 represents black and 100 represents white), a* for the green–red axis (where −a* denotes greenness and +a* denotes redness), and b* for the blue–yellow axis (where −b* denotes blueness and +b* denotes yellowness) [25].

The spread ratio, diameter, and thickness of the cookies were measured using the method described by Mudgil and Barak [19]. To measure the diameter, six cookies were arranged edge to edge, and the average diameter was calculated. The thickness was determined by stacking six cookies, and the average thickness was calculated using a caliper. The spread ratio of the cookies was calculated using the following equation:

Spread ratio = Average of diameter (mm)/Average of thickness (mm)
(6)

The moisture content of cookies was determined following the previous study [26], using an infrared moisture analyzer (FD-610 Kett Electric Laboratory, Tokyo, Japan). Briefly, five grams of ground cookies were accurately weighed and placed on an aluminum dish with a cover before being dried. The processing temperature for moisture analysis was set at 140 °C for 5 min. The results were reported as the percentage of moisture content in the cookies. 

The textural profile of cookies, including hardness and fracturability, was determined using a texture analyzer in compression mode with an HDP/3PB probe (Lloyd Instruments/Ametek TA1 Texture Analyzer, AMETEK (GB) Ltd., West Sussex, UK), as described in previous studies [25]. The hardness of the cookies, indicated by the maximum peak force required for breakage, and the fracturability, determined by the distance (mm) of the first significant break peak in the texture profile analysis curve, were analyzed. The compression test was conducted with a return to start cycle, a pretest speed of 1.0 mm/s, a test speed of 2.0 mm/s, a post-test speed of 10 mm/s, and a distance of 5.0 mm.

### 2.11. Sensory Analysis

The sensory evaluation of the cookies involved 50 untrained panelists, who were both male and female, aged between 18 and 50 years, and did not have any sensory impairments or food allergies. These panelists were regular consumers of bakery products. The study received approval from the Office of the Faculty of Dentistry/Faculty of Pharmacy, Mahidol University Institutional Review Board (COE.No.MU-DT/PY-IRB 2023/015.0404 Project No. 2023/PY042) and was conducted in accordance with the laboratory’s ethical guidelines, with each panelist providing written informed consent. To prevent bias, the samples were assigned unique 3-digit codes, and their serving orders were randomized using software. Mouth rinsing with plain water was performed between samples. The sensory attributes of the cookies, including appearance, color, aroma, taste, texture, and overall acceptability, were evaluated using a 9-point hedonic scale, with 1 indicating “extremely dislike”, 5 indicating “neither like nor dislike”, and 9 indicating “like extremely”.

### 2.12. Statistical Analysis

All experiments were conducted in triplicate, unless otherwise stated. Statistical analysis was performed using one-way analysis of variance (ANOVA), and the post hoc comparison was made using Duncan’s test. The area under the curve (AUC) was calculated using the trapezoidal rule. The significance level for all analyses was set at *p* < 0.05. The statistical software used for the analysis was IBM SPSS version 22.0 (International Business Machines Corporation, Armonk, NY, USA).

## 3. Results and Discussion

**Figure 1:**
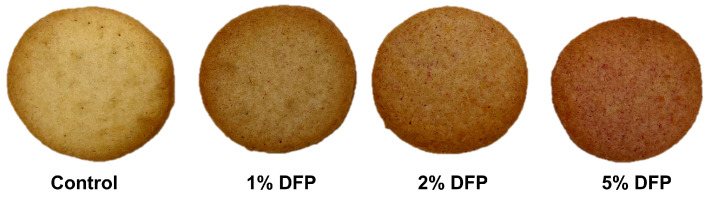
Photographs of cookies made with raw cookie dough containing dragon fruit peel at 1–5% (*w*/*w*).

Based on our preliminary investigation, we found that the viscosity of the cookie dough significantly increased when the concentration of DFP was increased beyond 5% (*w*/*w*). This increase in viscosity impeded the even spreading of the dough, leading to unevenness and lumpiness in the baked cookies due to inadequate heat penetration. As a result, we determined that the optimal range for incorporating DFP into cookies was between 1% and 5% (*w*/*w*). Within this range, the dough retained the desired consistency, which enabled it to be evenly spread, resulting in uniformly baked cookies with minimal lumpiness. The appearance of cookies produced from DFP is shown in Figure 1. As demonstrated in Table 2, the addition of DFP in varying concentrations to cookies resulted in a concentration-dependent increase in both TPC and TBC. The TPC increased from 173.48 mg GAE/100 g in the control cookie to 500.88 mg GAE/100 g in the 5% DFP cookie, while the TBC increased from 0.11 mg BE/100 g in the control cookie to 8035.71 mg BE/100 g in the 5% DFP cookie. Correspondingly, FRAP values for cookies containing 1%, 2%, and 5% DFP were 1.19-, 1.60-, and 2.16-fold higher than that of the control, respectively, indicating an increase in antioxidant activity. These results suggest that the addition of DFP to cookies can significantly enhance their phytochemical content and antioxidant capacity. The observed increase in phytochemical compounds and antioxidant capacity in cookies may be ascribed to the high levels of phytochemicals found in DFP, notably betanin, gallic acid, chlorogenic acid, syringic acid, coumaric acid, and ferulic acid. These compounds are well known for their potent antioxidant properties and have been reported to exhibit a wide range of health benefits, including anti-inflammatory, antimicrobial, and anticancer effects [17]. 

These findings are consistent with previous reports demonstrating that the addition of DFP to various food products, including mantou, noodles, bread, and cookies, can increase betacyanin and polyphenol content, as well as antioxidant capacity [17,18]. 

Cookies enriched with DFP at varying concentrations (1%, 2%, and 5%) exhibited notable reductions in the production of MDA (Table 2), a reactive aldehyde generated as a result of lipid peroxidation. Specifically, we observed dose-dependent reductions in MDA production, with the highest concentration of DFP leading to a remarkable 42.3% decrease in MDA levels. Notably, dietary fats exposed to high temperatures are known to generate high levels of oxidized compounds, including MDA. The accumulation of such compounds can increase lipid peroxidation products and lipophilic carbonyl compounds in blood and tissues, leading to reduced protein functionality, elevated production of reactive metabolites, oxidative modification of LDL, inflammation, and the initiation of atherosclerotic lesions and endothelial dysfunction [27,28]. Our findings suggest that the incorporation of DFP, which contains phytochemical compounds and dietary fibers, can effectively impede the formation of lipid peroxidation and its toxic by-products in baked products, potentially mitigating adverse health outcomes. The underlying mechanisms of DFP’s lipid-peroxidation inhibitory effects may involve the increased binding of free water content and the scavenging of free radicals. For instance, betanin, a pigment present in DFP, has been reported to inhibit lipid peroxidation in meat, possibly by reducing the concentration of reactive oxygen species [29]. Additionally, the high hydration properties of the dietary fibers present in DFP allow them to form a pseudoplastic barrier by binding with free water in the food system [18,30,31]. This can slow down the movement of free water molecules and reduce the extent of lipid peroxidation [32,33]. In addition to the potential health benefits, incorporating DFP into cookies may also prolong their shelf life by preventing fat-induced rancidity, thus improving the overall quality of the product.

**Figure 2:**
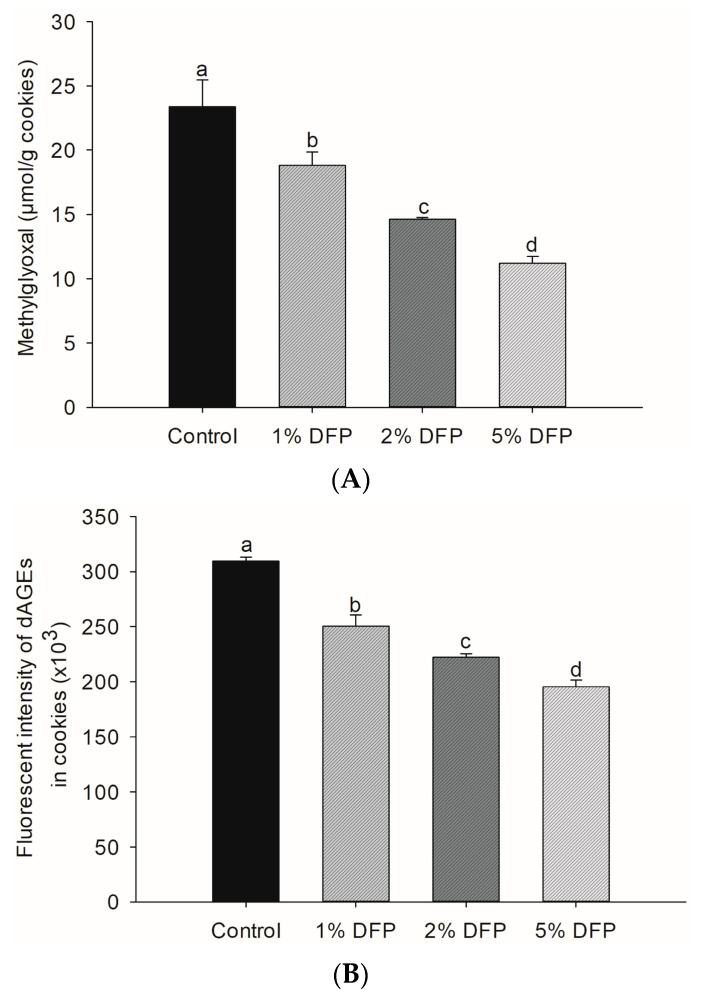
The effect of dragon fruit peel powder (DFP) used to fortify raw cookie dough on (**A**) the level of methylglyoxal and (**B**) dietary advanced glycation products (dAGEs). The results are presented as mean ± SEM, *n* = 3. Statistically significant differences in mean values are denoted by different letters, with a significance level set at *p* < 0.05.

MGO is a highly reactive compound that can be generated in food during various stages of processing, cooking, and storage. This occurs when the amino group of proteins reacts with the carbonyl group of reducing sugars in the presence of heat. These reactions result in the structural modification of proteins and the formation of dAGEs, which are formed through a series of intricate mechanisms such as caramelization, the Maillard reaction, and lipid peroxidation [5,34]. Our study findings revealed the promising potential of DFP in reducing the levels of MGO (Figure 2A) and dAGEs (Figure 2B) in cookies. By adding DFP at varying concentrations ranging from 1% to 5%, we observed a substantial reduction in MGO content, with the 5% DFP group exhibiting the most significant decrease of up to 52.1%, followed by the 2% and 1% DFP groups. Moreover, we observed a noteworthy inhibitory effect of DFP against dAGE formation in cookies, with the 5% DFP group demonstrating the highest efficacy in reducing dAGE formation by up to 36.9%, followed by the 2% and 1% DFP groups.

The results indicate that the incorporation of DFP into cookies may be a promising approach to reduce the formation of harmful compounds associated with thermal food processing and storage. The ability of DFP to inhibit the formation of MGO and dAGEs is attributed to the presence of its natural bioactive compounds, such as phenolics, betacyanins, and pectin [11,35]. Emerging scientific evidence suggests that phenolic compounds and betanin are capable of directly trapping intermediate glycation reactive compounds, such as MGO, and reducing the formation of AGE products, such as *N*^ε^-(carboxymethyl) lysine [11,36]. Pectin, on the other hand, has been shown to effectively inhibit the formation of AGEs in a BSA-galactose system [35]. Furthermore, DFP is also able to inhibit the formation of MGO and dAGEs by capturing reactive dicarbonyl compounds, scavenging free radicals, and chelating metal ions in heat-induced non-enzymatic browning reactions [11]. Notably, dietary fibers naturally present in DFP are known to increase the availability of water and dilute reactants in the aqueous phase, ultimately reducing the formation of dAGEs [33,35]. The dietary fibers also interfere with non-enzymatic browning reactions by interacting with amino acids, which helps prevent the formation of dAGEs during baking [31,32,33]. 

**Figure 3:**
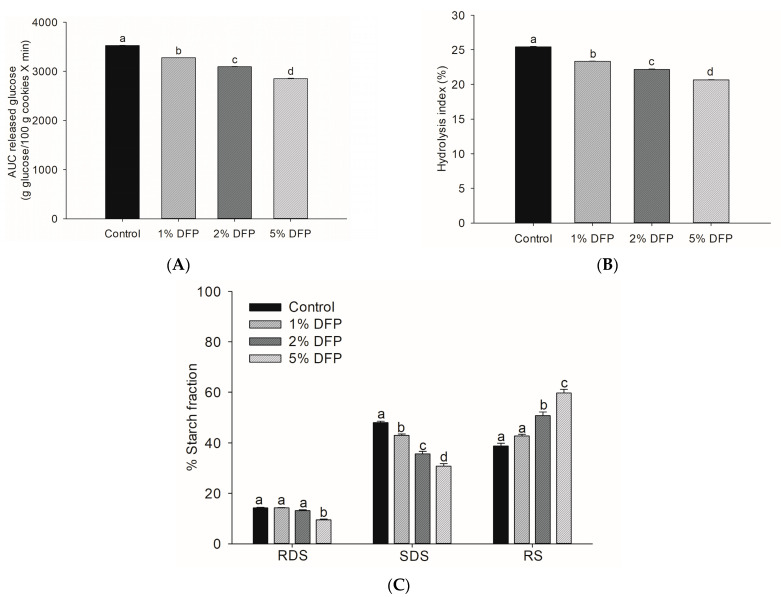
The area under the curve (AUC) of glucose release (**A**), hydrolysis index (**B**), and starch fractions (**C**) of cookies fortified with dragon fruit peel powder (DFP) in raw cookie dough during in vitro digestion. The results are presented as mean ± SEM, *n* = 3. Statistically significant differences in mean values are denoted by different letters, with a significance level set at *p* < 0.05. RDS: rapidly digestible starch; SDS: slowly digestible starch; RS: resistant starch.

Incorporating 5% DFP into cookies resulted in a remarkable reduction in glucose released during simulated digestion (Figure 3A), as indicated by a significant decrease in the area under the glucose release curve compared to the control cookie (*p* < 0.05). This decrease in starch digestibility rate, which was 18.9%, is consistent with our earlier study demonstrating the suppressive effect of DFP on starch digestibility when added to wheat flour [18]. The underlying mechanism for this effect is believed to be due to the presence of dietary fibers and phytochemicals in DFP, which alter starch properties by interfering with the formation of starch gel and chains, increasing onset temperature, decreasing gelatinization enthalpy, and disrupting the dissolution of the crystalline structures of starch granules [18]. Dietary fibers have been reported to alter the starch system by trapping starch granules and increasing viscosity, which reduces enzyme accessibility to starch [37]. Furthermore, phenolic compounds in DFP may interact with starch granules and digestive enzymes, leading to limited starch digestion [36,38]. To evaluate the nutritional properties of DFP cookies, we conducted an analysis of the hydrolysis index (HI) and predicted glycemic index (pGI), crucial indicators for estimating the physiological glycemic response in the in vivo system [39,40]. With the addition of 1%, 2%, and 5% of DFP, the HI percentage of DFP cookies was lower than that of the control by 8.6%, 12.9%, and 19.0%, respectively (Figure 3B). Remarkably, DFP significantly reduced the glycemic index in cookies by decreasing pGI from 53.68 in the control to 52.53 in 1% DFP, 51.89 in 2% DFP, and 51.04 in 5% DFP (Table 2). These findings indicate that DFP can be employed to prepare functional cookies, resulting in reduced starch digestibility rates and low-glycemic-index foods that can become a healthier bakery option for consumers. From a nutritional standpoint, dietary starches are classified into three primary fractions based on their in vitro digestibility: rapidly digestible starch (RDS), slowly digestible starch (SDS), and resistant starch (RS) [39]. RS, or undigested starch, is particularly beneficial to health as it remains undigested after 120 min, thus controlling the postprandial glycemic response [41]. Significantly, the addition of DFP to cookies increased the amount of undigested starch while decreasing the RDS and SDS compared to cookies without DFP (Figure 3C). A similar outcome was reported in a previous study where cookies treated with passion fruit peel flour containing high levels of dietary fibers and bioactive compounds demonstrated a lower hydrolysis percentage and reduced RDS content [3]. Research has shown that consuming a diet containing a reduced fraction of SDS and an increased proportion of undigested starch can yield several health benefits, such as improved glycemic control and insulin sensitivity [41,42]. Consequently, the incorporation of DFP in cookies can substantially lower the rate of starch digestibility and augment the content of resistant starch, thereby producing low-glycemic-index foods that hold promise as potential health-promoting dietary options. Further investigations are required to assess the acute effects of DFP cookies on postprandial glycemic response in human subjects.

Table 3 provides a comprehensive overview of the physical characteristics of cookies supplemented with DFP. The addition of DFP had a significant influence on the cookies’ color, spread ratio, moisture content, and texture profiles.

The results indicated a noteworthy increase in the redness (a*) of the cookies, while the yellowness (b*) declined significantly (*p* < 0.05). This effect can be attributed to the presence of betacyanin pigments found in the red peels of the DFP [18,43]. Interestingly, the incorporation of 2% or 5% DFP resulted in a statistically significant reduction in the spread ratio of cookies. This outcome can be attributed to the pronounced water-binding capacity of DFP, which led to an increase in the thickness and a decrease in the diameter of the cookies [18]. We suggest that the incorporation of DFP into the dough mixture resulted in the absorption of water, which elevated the dough’s viscosity and consistency. Consequently, the insufficient sugar dissolves in the dough system, leading to a decrease in the diameter of dough expansion but an increase in cookie thickness during baking [44,45]. This finding aligns with previous studies, which have reported that high hydration properties of ingredients tend to reduce the width and spread factor of cookies [45]. Moreover, the moisture content of the cookies significantly increased when 2% or more DFP was added to the formulation [46]. With respect to the texture profile analysis, the hardness of the cookies decreased significantly, while the fracturability increased with an increase in the DFP level. This phenomenon can be attributed to gluten dilution and the high-water absorption capacity of DFP, which hinder gluten development upon the addition of dietary fibers [45,46]. These findings provide insight into the potential of DFP as a functional ingredient in bakery products, offering not only desirable color attributes but also improving texture and moisture profiles.

The sensory acceptability of cookie characteristics, including appearance, color, odor, taste, texture, hardness, and overall acceptance, were evaluated for cookies containing DFP, as shown in Table 4. The acceptability scores for appearance, odor, texture, and hardness were not significantly different from those of the control group, except for the 5% DFP addition. Regarding color and taste, the 2% DFP addition had the highest score, while the 5% DFP addition had the lowest score. These findings could be attributed to the darker color and slightly sour, as well as bitter taste derived from DFP, as previously reported [15,43]. In this study, the overall acceptability score of DFP-treated cookies was greater than 7, which is indicative of high sensory acceptance [47]. Moreover, the highest acceptance score was observed for the 2% DFP addition, indicating the high acceptability of DFP addition in cookies. Therefore, it appears feasible to manufacture a functional cookie containing dietary fiber and phytochemicals with an acceptable sensory quality with the addition of DFP. However, further studies are necessary to clarify the impact of DFP on physical properties, sensory qualities, and acceptability during storage.

## 4. Conclusions

Incorporating dragon fruit peel powder (DFP) in raw cookie dough can enhance the nutritional and functional properties of cookies. The addition of DFP can increase the bioactive phytochemical content and antioxidant capacity of cookie products, decrease levels of heat-generated food toxicants, and reduce starch digestibility. Although cookies enriched with DFP showed alterations in their physical properties, incorporating up to 2% DFP did not affect the overall acceptability of the cookies. This innovative approach offers a promising opportunity for the food industry to increase the value of waste materials generated during fruit processing and create healthier food products with improved sustainability.

## Figures and Tables

**Table 1:** Cookie recipes fortified with dragon fruit peel powder (DFP).

Ingredients	Composition of Cookies (%*w/w* of the Weight of Raw Cookie Dough)			
	Control	1% DFP	2% DFP	5% DFP
Wheat flour	34.2	34.2	34.2	34.2
Skim milk powder	5.9	5.9	5.9	5.9
Sugar	20.0	20.0	20.0	20.0
Egg	13.7	13.7	13.7	13.7
Vanilla flavor	0.2	0.2	0.2	0.2
Unsalted butter	25.1	25.1	25.1	25.1
Baking soda	0.9	0.9	0.9	0.9
DFP	0.0	1.0	2.0	5.0

**Table 2:** Effects of adding dragon fruit peel powder (DFP) to raw cookie dough on total phenolic content (TPC), total betacyanin content (TBC), ferric-reducing antioxidant power (FRAP) activity, lipid peroxidation, and predicted glycemic index (pGI).

Experiments	TPC	TBC	FRAP	Lipid Peroxidation	pGI
	(mg GAE/100 g Cookie)	(mg BE/100 g Cookie)	(μmol FeSO_4_ Equivalent/100 g Cookie)	(μmol MDA Equivalent/100 g Cookie)	
Control	173.48 ± 2.45 ^a^	0.11 ± 0.02 ^a^	752.75 ± 8.47 ^a^	6.38 ± 0.15 ^a^	53.68 ± 0.1 ^a^
1% DFP	239.77 ± 8.32 ^b^	4519.48 ± 29.99 ^b^	894.87 ± 3.60 ^b^	4.90 ± 0.06 ^b^	52.53 ± 0.3 ^b^
2% DFP	263.37 ± 2.87 ^c^	6087.66 ± 75.42 ^c^	1207.24 ± 10.91 ^c^	3.94 ± 0.08 ^c^	51.89 ± 0.2 ^c^
5% DFP	500.88 ± 3.45 ^d^	8035.71 ± 37.26 ^d^	1624.44 ± 12.80 ^d^	2.66 ± 0.02 ^d^	51.04 ± 0.2 ^d^

The values are presented as means ± standard error of the mean (SEM), *n* = 3. Means with a different superscript indicate a significant difference (*p* < 0.05). GAE—gallic acid equivalent; BE—betanin equivalent; MDA—malondialdehyde.

**Table 3:** The physical characteristics of cookies fortified with dragon fruit peel powder (DFP).

Experiments	Control	1% DFP	2% DFP	5% DFP
Color				
L*	47.86 ± 4.31 ^a^	53.48 ± 3.00 ^a^	53.27 ± 0.29 ^a^	48.78 ± 0.83 ^a^
a*	6.03 ± 0.20 ^a^	7.24 ± 0.49 ^b^	8.26 ± 0.23 ^ab^	9.10 ± 0.25 ^c^
b*	36.04 ± 0.32 ^a^	34.73 ± 0.44 ^ab^	32.66 ± 2.19 ^ab^	31.60 ± 0.56 ^b^
Spread ratio	9.10 ± 0.25 ^a^	8.26 ± 0.23 ^ab^	7.24 ± 0.49 ^b^	6.03 ± 0.20 ^c^
Moisture (%)	5.13 ± 0.02 ^a^	5.07 ± 0.03 ^a^	5.40 ± 0.05 ^b^	5.80 ± 0.05 ^c^
Texture profiles				
Hardness (N)	34.09 ± 1.93 ^a^	25.28 ± 1.93 ^b^	19.83 ± 1.19 ^c^	17.66 ± 1.27 ^c^
Fracturability (mm)	1.01 ± 0.06 ^a^	1.05 ± 0.10 ^a^	2.04 ± 0.20 ^b^	2.26 ± 0.16 ^b^

The values are presented as means ± standard error of the mean (SEM), *n* = 3. Means with a different superscript in each row indicate a significant difference (*p* < 0.05).

**Table 4:** Sensory evaluation of cookies fortified with dragon fruit peel powder (DFP).

Sensory Characteristics	Control	1% DFP	2% DFP	5% DFP
Appearance	7.16 ± 0.17 ^a^	7.12 ± 0.19 ^a^	7.32 ± 0.16 ^b^	6.80 ± 0.20 ^c^
Color	7.38 ± 0.15 ^a^	7.26 ± 0.17 ^a^	7.48 ± 0.14 ^a^	6.54 ± 0.18 ^b^
Odor	7.34 ± 0.17 ^a^	7.28 ± 0.19 ^a^	7.22 ± 0.17 ^a^	6.9 ± 0.21 ^a^
Taste	7.36 ± 0.17 ^a^	7.26 ± 0.10 ^ab^	7.50 ± 0.19 ^a^	6.92 ± 0.08 ^b^
Texture	7.20 ± 0.21 ^a^	7.36 ± 0.19 ^a^	7.48 ± 0.17 ^b^	7.02 ± 0.20 ^c^
Hardness	7.92 ± 0.14 ^a^	7.80 ± 0.18 ^ab^	7.80 ± 0.15 ^ab^	7.56 ± 0.15 ^b^
Overall acceptability	7.60 ± 0.15 ^a^	7.56 ± 0.15 ^a^	7.74 ± 0.15 ^a^	7.16 ± 0.16 ^b^

The values are presented as means ± standard error of the mean (SEM), *n* = 50. Means with a different superscript in each row indicate a significant difference (*p* < 0.05).

## Data Availability

Data are contained within the article.

## References

[1] Arribas-Lorenzo G., Morales F.J. (2010). Analysis, distribution, and dietary exposure of glyoxal and methylglyoxal in cookies and their relationship with other heat-induced contaminants. J. Agric. Food Chem..

[2] Carnell S., Benson L., Gibson E.L., Mais L.A., Warkentin S. (2017). Caloric compensation in preschool children: Relationships with body mass and differences by food category. Appetite.

[3] Ning X., Wu J., Luo Z., Chen Y., Mo Z., Luo R., Bai C., Du W., Wang L. (2021). Cookies fortified with purple passion fruit epicarp flour: Impact on physical properties, nutrition, in vitro starch digestibility, and antioxidant activity. Cereal Chem..

[4] Kuzan A. (2021). Toxicity of advanced glycation end products (Review). Biomed. Rep..

[5] Nowotny K., Schröter D., Schreiner M., Grune T. (2018). Dietary advanced glycation end products and their relevance for human health. Ageing Res. Rev..

[6] Eder K., Keller U., Hirche F., Brandsch C. (2003). Thermally oxidized dietary fats increase the susceptibility of rat LDL to lipid peroxidation but not their uptake by macrophages. J. Nutr..

[7] Sergi D., Boulestin H., Campbell F.M., Williams L.M. (2021). The role of dietary advanced glycation end products in metabolic dysfunction. Mol. Nutr. Food Res..

[8] Wang S., Zheng L., Zheng X., Yang Y., Xiao D., Zhang H., Ai B., Sheng Z. (2022). Chitosan inhibits advanced glycation end products formation in chemical models and bakery food. Food Hydrocoll..

[9] Zhang X., Chen F., Wang M. (2014). Antioxidant and antiglycation activity of selected dietary polyphenols in a cookie model. J. Agric. Food Chem..

[10] Gao J., Sun Y., Li L., Zhou Q., Wang M. (2020). The antiglycative effect of apple flowers in fructose/glucose-BSA models and cookies. Food Chem..

[11] Song Q., Liu J., Dong L., Wang X., Zhang X. (2021). Novel advances in inhibiting advanced glycation end product formation using natural compounds. Biomed. Pharmacother..

[12] Torres J.D., Dueik V., Carré D., Bouchon P. (2019). Effect of the addition of soluble dietary fiber and green tea polyphenols on acrylamide formation and in vitro starch digestibility in baked starchy matrices. Molecules.

[13] Dikeman C.L., Murphy M.R., Fahey G.C. (2006). Dietary fibers affect viscosity of solutions and simulated human gastric and small intestinal digesta. J. Nutr..

[14] Xiao H., Lin Q., Liu G.-Q., Wu Y., Tian W., Wu W., Fu X.-J. (2011). Effect of green tea polyphenols on the gelatinization and retrogradation of rice starches with different amylose contents. J. Med. Plant Res..

[15] Bakar J., Shu C.E., Muhammad S., Hashim D.M., Noranizan A. (2011). Physico-chemical characteristics of red pitaya (*Hylocereus polyrhizus*) peel. Int. Food Res. J..

[16] Cheok C.Y., Mohd Adzahan N., Abdul Rahman R., Zainal Abedin N.H., Hussain N., Sulaiman R., Chong G.H. (2018). Current trends of tropical fruit waste utilization. Crit. Rev. Food Sci. Nutr..

[17] Jiang H., Zhang W., Li X., Shu C., Jiang W., Cao J. (2021). Nutrition, phytochemical profile, bioactivities and applications in food industry of pitaya (*Hylocereus* spp.) peels: A comprehensive review. Trends Food Sci. Technol..

[18] Chumroenvidhayakul S., Thilavech T., Abeywardena M., Adisakwattana S. (2022). Investigating the impact of dragon fruit peel waste on starch digestibility, pasting, and thermal properties of flours used in Asia. Foods.

[19] Mudgil D., Barak S., Khatkar B.S. (2017). Cookie texture, spread ratio and sensory acceptability of cookies as a function of soluble dietary fiber, baking time and different water levels. LWT.

[20] Adisakwattana S., Intrawangso J., Hemrid A., Chanathong B., Mäkynen K. (2012). Extracts of edible plants inhibit pancreatic lipase, cholesterol esterase and cholesterol micellization, and bind bile acids. Food Technol. Biotechnol..

[21] Benzie I.F.F., Strain J.J. (1996). The ferric reducing ability of plasma (FRAP) as a measure of “antioxidant power”: The FRAP assay. Anal. Biochem..

[22] Chusak C., Thilavech T., Henry C.J., Adisakwattana S. (2018). Acute effect of *Clitoria ternatea* flower beverage on glycemic response and antioxidant capacity in healthy subjects: A randomized crossover trial. BMC Complement. Altern. Med..

[23] Goñi I., Garcia-Alonso A., Saura-Calixto F. (1997). A starch hydrolysis procedure to estimate glycemic index. Nutr. Res..

[24] Thilavech T., Ngamukote S., Belobrajdic D., Abeywardena M., Adisakwattana S. (2016). Cyanidin-3-rutinoside attenuates methylglyoxal-induced protein glycation and DNA damage via carbonyl trapping ability and scavenging reactive oxygen species. BMC Complement. Altern. Med..

[25] Bukolt K., Ramirez N., Saenz A., Mirza K., Bhaduri S., Navder K. (2019). Effect of low glycemic index stevia-benefiber sweetener on the physical, textural and sensory qualities of oatmeal raisin cookies. J. Food Process. Technol..

[26] Laganà V., Giuffrè A.M., De Bruno A., Poiana M. (2022). Formulation of biscuits fortified with a flour obtained from bergamot by-products (*Citrus bergamia*, Risso). Foods.

[27] Kanner J. (2007). Dietary advanced lipid oxidation end products are risk factors to human health. Mol. Nutr. Food Res..

[28] Staprans I., Hardman D.A., Pan X.M., Feingold K.R. (1999). Effect of oxidized lipids in the diet on oxidized lipid levels in postprandial serum chylomicrons of diabetic patients. Diabetes Care.

[29] Vieira Teixeira da Silva D., Dos Santos Baião D., de Oliveira Silva F., Alves G., Perrone D., Mere Del Aguila E., Paschoalin V.M.F. (2019). Betanin, a natural food additive: Stability, bioavailability, antioxidant and preservative ability assessments. Molecules.

[30] Yemenicioğlu A., Farris S., Turkyilmaz M., Gulec S. (2020). A review of current and future food applications of natural hydrocolloids. Int. J. Food Sci. Technol..

[31] Liu J., Fang C., Luo Y., Ding Y., Liu S. (2019). Effects of konjac oligo-glucomannan on the physicochemical properties of frozen surimi from red gurnard (*Aspitrigla cuculus*). Food Hydrocoll..

[32] Zhang Q., Wang Y., Fu L. (2020). Dietary advanced glycation end-products: Perspectives linking food processing with health implications. Compr. Rev. Food Sci. Food Saf..

[33] Chaouch M.A., Hafsa J., Rihouey C., Le Cerf D., Majdoub H. (2016). Effect of extraction conditions on the antioxidant and antiglycation capacity of carbohydrates from *Opuntia robusta* cladodes. Int. J. Food Sci. Technol..

[34] Han J., Tan C., Wang Y., Yang S., Tan D. (2015). Betanin reduces the accumulation and cross-links of collagen in high-fructose-fed rat heart through inhibiting non-enzymatic glycation. Chem. Biol. Interact..

[35] Passos C.P., Ferreira S.S., Serôdio A., Basil E., Marková L., Kukurová K., Ciesarová Z., Coimbra M.A. (2018). Pectic polysaccharides as an acrylamide mitigation strategy—Competition between reducing sugars and sugar acids. Food Hydrocoll..

[36] Mirmiran P., Houshialsadat Z., Gaeini Z., Bahadoran Z., Azizi F. (2020). Functional properties of beetroot (*Beta vulgaris*) in management of cardio-metabolic diseases. Nutr. Metab..

[37] Repin N., Cui S.W., Goff H.D. (2018). Impact of dietary fibre on in vitro digestibility of modified tapioca starch: Viscosity effect. Bioact. Carbohydr. Diet. Fibre.

[38] Zhu F. (2015). Interactions between starch and phenolic compound. Trends Food Sci. Technol..

[39] Englyst H.N., Kingman S.M., Cummings J.H. (1992). Classification and measurement of nutritionally important starch fractions. Eur. J. Clin. Nutr..

[40] Lal M.K., Singh B., Sharma S., Singh M.P., Kumar A. (2021). Glycemic index of starchy crops and factors affecting its digestibility: A review. Trends Food Sci. Technol..

[41] Bojarczuk A., Skąpska S., Mousavi Khaneghah A., Marszałek K. (2022). Health benefits of resistant starch: A review of the literature. J. Funct. Foods.

[42] Zhang G., Hamaker B.R. (2009). Slowly digestible starch: Concept, mechanism, and proposed extended glycemic index. Crit. Rev. Food Sci. Nutr..

[43] Ho L.-H., Abdul Latif N.W.B. (2016). Nutritional composition, physical properties, and sensory evaluation of cookies prepared from wheat flour and pitaya (*Hylocereus undatus*) peel flour blends. Cogent Food Agric..

[44] Becker F.S., Damiani C., de Melo A.A.M., Borges P.R.S., de Barros Vilas Boas E.V. (2014). Incorporation of buriti endocarp flour in gluten-free whole cookies as potential source of dietary fiber. Plant Foods Hum. Nutr..

[45] Mancebo C.M., Rodríguez P., Martínez M.M., Gómez M. (2018). Effect of the addition of soluble (nutriose, inulin and polydextrose) and insoluble (bamboo, potato and pea) fibres on the quality of sugar-snap cookies. Int. J. Food Sci..

[46] Palaniappan A., Abirami A., Anbuvahini N., Kumaran T., Naresh M., Malathi D., Antony U. (2015). Physicochemical properties of cookies enriched with xylooligosaccharides. Food Sci. Technol. Int..

[47] Everitt M., Barbosa-Cánovas G., Mortimer A., Lineback D., Spiess W., Buckle K., Colonna P. (2009). CHAPTER 8—Consumer-Targeted Sensory Quality. Global Issues in Food Science and Technology.

